# Niosome-Based Approach for In Situ Gene Delivery to Retina and Brain Cortex as Immune-Privileged Tissues

**DOI:** 10.3390/pharmaceutics12030198

**Published:** 2020-02-25

**Authors:** Nuseibah AL Qtaish, Idoia Gallego, Ilia Villate-Beitia, Myriam Sainz-Ramos, Tania Belén López-Méndez, Santiago Grijalvo, Ramón Eritja, Cristina Soto-Sánchez, Gema Martínez-Navarrete, Eduardo Fernández, Gustavo Puras, José Luis Pedraz

**Affiliations:** 1 NanoBioCel group, University of the Basque Country (UPV/EHU), E-01006 Vitoria-Gasteiz, Spain; nusaiba.qtaish@gmail.com (N.A.Q.); idoiagallego@hotmail.com (I.G.); aneilia.villate@ehu.eus (I.V.-B.); myri.2694@gmail.com (M.S.-R.); tblopez01@gmail.com (T.B.L.-M.); 2 Networking Research Centre of Bioengineering, Biomaterials and Nanomedicine (CIBER-BBN), E-01006 Vitoria-Gasteiz, Spain; 3 Networking Research Centre of Bioengineering, Biomaterials and Nanomedicine (CIBER-BBN), E-08034 Barcelona, Spain; sgrgma@cid.csic.es (S.G.); recgma@cid.csic.es (R.E.); 4 Institute for Advanced Chemistry of Catalonia, (IQAC-CSIC), E-08034 Barcelona, Spain; 5 Neuroprothesis and Neuroengineering Research Group, Miguel Hernández University, E-03202 Elche, Spain; csoto@goumh.umh.es (C.S.-S.); gemamartineznavarrete@gmail.com (G.M.-N.); e.fernandez@umh.es (E.F.); 6 Networking Research Centre for Bioengineering, Biomaterials and Nanomedicine (CIBER-BBN), E-03202 Elche, Spain

**Keywords:** gene delivery, non-viral vectors, niosomes, brain, retina

## Abstract

Non-viral vectors have emerged as a promising alternative to viral gene delivery systems due to their safer profile. Among non-viral vectors, recently, niosomes have shown favorable properties for gene delivery, including low toxicity, high stability, and easy production. The three main components of niosome formulations include a cationic lipid that is responsible for the electrostatic interactions with the negatively charged genetic material, a non-ionic surfactant that enhances the long-term stability of the niosome, and a helper component that can be added to improve its physicochemical properties and biological performance. This review is aimed at providing recent information about niosome-based non-viral vectors for gene delivery purposes. Specially, we will discuss the composition, preparation methods, physicochemical properties, and biological evaluation of niosomes and corresponding nioplexes that result from the addition of the genetic material onto their cationic surface. Next, we will focus on the in situ application of such niosomes to deliver the genetic material into immune-privileged tissues such as the brain cortex and the retina. Finally, as future perspectives, non-invasive administration routes and different targeting strategies will be discussed.

## 1. Introduction

It has been a long journey, with promising expectations and serious setbacks since gene therapy was referred as a potential strategy to face monogenetic disorders 45 years ago, until nowadays, where this advanced therapy is considered a realistic, although still uncommon, medical option for the treatment of both inherited and acquired human diseases [1]. The knowledge gained on the molecular basis of genetic diseases along with recent advances in different research areas, such as biotechnology or nanomedicine, have contributed to increasing the number of clinical trials based on gene therapy up to around 2000 (http://www.abedia.com/wiley/). Such interest has accelerated the research investment of many companies involved in the development of gene therapy-based drugs, and consequently, it is expected that the therapeutic armamentarium will soon increase [2].

The main concept of gene therapy is quite simple and basically relies on the incorporation of enough exogenous genetic material into a specific target cell in a safe way to modulate protein expression related to the development of diseases that cannot be faced with conventional treatments [3]. More specifically, therapeutic genetic material can be supplied to cells with genetically modified viruses (virotherapy) due to their natural ability to infect cells [4]. This approach is particularly interesting to selectively infect and kill cancer cells, although the use of biological agents such as infecting viruses for medical applications raises relevant safety concerns [5]. Another alternative to enhance the expression of a specific protein whose low levels accelerate the development of certain diseases is through the administration of bacterial plasmid DNA (pDNA) [6]. This strategy is normally applied for the treatment of genetic diseases that follow an autosomic recessive inheritance pattern. However, the main drawbacks of plasmid administration include the immune response generated against the bacterial elements [7] and, in some cases, the big size of the plasmid that decreases transfection efficiency process [8]. To minimize such disadvantages, unmethylated cytosine-phosphate-guanine (CpG) dinucleotides from bacterial origin and other not relevant sequences related to the origin of replication and the resistance to antibiotics have been removed from conventional plasmids resulting in minicircle DNAs (mcDNAs), which reduce immunogenic response and enhance transfection efficiency, allowing a sustained expression of the therapeutic gene (transgene) [9]. Other different approach include the administration of exogenous genetic material in the form of small interfering RNA (siRNA), or aptamers to inhibit protein expression by different mechanisms at a post-transcriptional level, or the synthesis of antisense oligonucleotides (ASOs) that can regulate the expression of both precursor RNA (pre-RNA) or mature RNA in the nucleus or cytosol, respectively [10,11,12,13]. These therapeutic oligonucleotides are very sensitive to enzymatic degradation, and therefore, the biomacromolecules must be stabilized with chemical modifications on their structure [11,14].

Normally, and in clear contrast to conventional drug-based therapies, marketed gene therapy products are designed to get long-lasting therapeutic benefits and focus their interest on rare and specific disorders that affect a reduced number of patients. In this sense, it is worth mentioning the case of the recently approved Milasen^®^ drug, which has been specifically designed for a single patient suffering from Batten disease [13]. The unusual characteristics of gene therapy-based drugs also raise ethical and social concerns related to the cost of such innovative treatments [2]. Some in vivo gene therapy products currently approved for human use are summarized in Table 1.

Another emerging strategy to deliver transgenes into the organism is through the extraction of cells from the patient, which after ex vivo genetic manipulation are implanted again into the organism [15,16]. In fact, recently, many ex vivo gene therapy products such as Zalmoxis, Zyntelgo, Invossa, Yeskarta, Kymriah and Strimvelis have been commercialized [17]. Such approaches use both retro and lentivirus vectors to transduce allogenic and autologous cells for the treatment of hematopoietic malignancies, osteoarthritis, or severe combined immunodeficiency diseases.

In addition to the previously described gene therapy approaches based on both gene supplementation and gene suppression strategies, recent advances on genome editing tools by CRISPR/Cas technology allow the correction of a specific mutation at a genomic level [18]. Due to the huge treatment possibilities of such revolutionary genome editing tools, the number of scientific publications in this area has considerably increased since 2014, and many clinical trials are underway, especially in cancer and pathological disorders of the blood and eye [19]. In any case, although highly promising, still some concerns mainly related to the delivery strategy, the possibility of permanent off target effects, or the efficiency to repair the mutation in a controlled manner need to be resolved before reaching the market [20]. In this sense, the new modified version of the CRISPR/Cas systems referred as “prime editing” holds great potential to promote the translation of this technology into clinical practice [21].

Although few gene therapy products are available for human use, there is no doubt that this market has significantly increased in the last few years. Consequently, it is reasonably estimated that some products that nowadays are under clinical trials evaluation will soon reach the clinical practice, which justify the optimism and financial investment of many biotechnology firms [1]. In any case, more research efforts need to be focused on the development of safe and efficient genetic material delivery systems to overcome the biological barriers that hamper the clinical application of gene therapy. This issue is particularly relevant for the treatment of diseases that affect to sensitive and immunologically isolated organs, such as brain and eye, where gene therapy-based drugs should be preferably administered by non-invasive administration routes.

## 2. Biological Barriers

To be active at the place of action, gene delivery systems need to overcome both extracellular and intracellular barriers for in vivo applications, while for ex vivo purposes, only intracellular barriers can hamper their final performance [22]. Extracellular biological barriers to overcome will depend mainly on the administration route, while intracellular barriers will differ according to the target cell.

### 2.1. Extracellular Barriers

**Figure 1:**
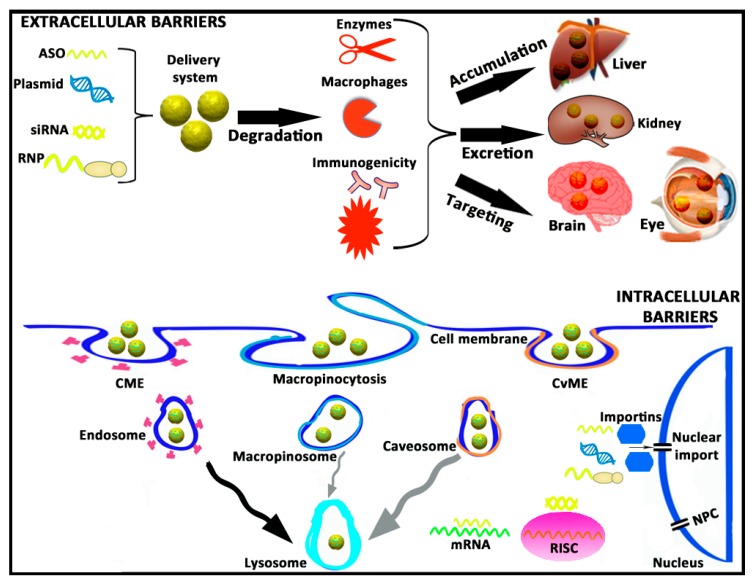
A brief schematic representation of both extracellular and intracellular barriers that genetic material needs to overcome during the transfection process. ASO, antisense oligonucleotide; siRNA, small interfering RNA; RNP, ribonucleoprotein; CME, clathrin-mediated endocytosis; CvME, caveolae-mediated endocytosis; RISC, RNA-induced silencing complex; mRNA, messenger RNA; NPC, nuclear pore complex.

From a practical point of view, the intravenous administration of gene therapy-based drugs represents a promising approach to face diseases that affect the liver due to the natural tendency to be accumulated in such an organ [23]. In addition, because there is not an absorption process, the bioavailability of drugs is 100%. Considering that cancer disease represents around 65% of current gene therapy clinical trials (http://www.abedia.com/wiley/) this route of administration is also interesting to treat disseminated cancer cells that affect many organs. However, despite these relevant advantages, its effect is highly hampered by the possible drug-induced hepatotoxicity [24] and by the relevant biological extracellular barriers that the genetic material needs to overcome [25]. Consequently, biomacromolecules such as ASOs, plasmids, siRNAs, or ribonucleoproteins (RNPs) are normally administered with different kinds of gene delivery systems (viral or non-viral vectors) specifically designed to make the process more efficient [26]. Usually, such biomacromolecules, in the “naked” form (without any gene delivery system or chemical modification) can be easily degraded immediately after administration by proteases and nucleases present in the blood. In addition, biomacromolecules can also be phagocytosed by macrophages, and the bacterial origin of their components can induce both cellular and humoral immune responses, which not only jeopardize their final performance but can also have deleterious effects on the safety profile after their administration [27]. In circulation, innate immune responses can occur through the activation of different kind of toll-like receptors (TLRs) by impurities such as endotoxins or by bacterial components such as CpG motifs present on the genetic material. Furthermore, an adaptive immune response can also be activated in the case of pre-existing immunity [28]. In the case of genetic material delivered by non-viral vectors, some physicochemical parameters such as zeta potential, particle size destruction, polydispersity index, or hydrophobicity/hydrophilicity balance can also contribute to the compatibility with the immune system [29]. Another relevant issue that needs to be considered is the natural tendency to accumulate in the liver after intravenous administration, and the fact that such biomacromolecules, due to their small size, below 5.5 nm, can be cleared quickly from systemic circulation through kidneys by renal excretion [30]. In any case, although genetic material remains stable in bloodstream without eliciting an immune response, the time required to reach therapeutic concentrations—the transfection process—can be even more challenging if isolated and immune-privileged organs such as brain and eye are the final target of the gene therapy treatment. In this case, additional extracellular barriers that protect the brain and eye from the rest of the organism such as the blood–brain barrier (BBB) and the blood–retinal barrier (BRB) need to be overcome [31,32]. Due to the critical obstacles that extracellular barriers represent, the design of effective and safe gene delivery systems for in vivo gene therapy represents a really stimulating task for the scientific community. A schematic representation of extracellular barriers is shown in Figure 1.

### 2.2. Intracellular Barriers

Once extracellular barriers have been overcome, biomacromolecules still need to reach sufficient amounts at cytoplasmic, or even nuclear levels, preferably only on the target cells, in order to be biologically active. Again, this intracellular trafficking is another arduous journey full of hurdles to beat for the genetic material [33]. First of all, negatively charged ASOs, plasmids, siRNAs, and RNPs are electrostatically repealed by the hydrophilic anionic proteins of the cell membrane, which jeopardizes their cellular uptake and posterior internalization process [34]. In the absence of antibodies or cell-specific ligands that promote a targeting effect, biomacromolecules can be internalized by different pathways, of which clathrin-mediated endocytosis (CME), macropinocytosis and caveolae-mediated endocytosis (CvME) are the most representative ones, forming the corresponding intracellular vesicles referred as endosomes, macropinosomes, and caveosomes, respectively [35]. Although there is not a unique consensus, and results in this research area are quite controversial, it is estimated that those endocytosis pathways are connected, in a great (CME) or less extension (CvME and macropinocytosis), to the lysosomes, where the acidic pH value degrades the genetic material. Consequently, the transfection process is strongly affected [36]. If biomacromolecules escape on time from the acidic environment of lysosomes, they still need to move quickly and properly through the cytosol to reach the RNA-induced silencing complex (RISC) in the case of siRNA, or the target mRNA in the case of ASOs that inactivate mature RNA. In the case of plasmids, RNPs, and some ASOs that act on pre-RNA, the impermeability of the nuclear membrane represents another hurdle to overcome, especially in quiescent and non-dividing cells [37,38]. Nuclear pore complexes (NPC) present on the nuclear membrane of cells with a small 9 nm channel diameter that prevents the entry into the nucleus of chemical compounds with a molecular weight over 45 kDa [38]. Other alternative to cross the nuclear membrane is through active mechanisms mediated mainly by importins of the cytoplasm that promote nuclear translocation [39]. Once inside the nucleus, or even before during the cytoplasmic trafficking, biomacromolecules need to be dissociated from gene delivery systems to get access into the transcriptional machinery of the target cell and produce the final biological effect [40]. A brief schematic representation of intracellular hurdles is shown in Figure 1.

## 3. Non-Viral Gene Delivery Systems

Classically, gene delivery systems designed to overcome biological barriers are classified as viral and non-viral vectors. Viruses, independently of their origin, have evolved over millions of years to gain access into host eukaryotic cells in order to shuttle their genetic cargo. Nowadays, recombinant viruses have been modified in the laboratory to reduce their pathogenic effect and to deliver the transgene of interest into target cells [41]. During the last few years, relevant improvements have been made mainly regarding their production methodology, safety profile, and genetic material packing capacity. However, their biological origin hampers the commercialization process by regulatory authorities, which clearly impacts on their final price [2,42]. In contrast, the non-viral vectors counterparts are classically recognized for their safety profile, higher packing capacity, and low cost of production [43]. In any case, non-viral vectors for plasmid-based gene therapy have not yet reached clinical practice, although research on this topic has quickly increased during the last few years, which has been especially motivated by the impact that CRISPR/Cas technology has had on scientific community and the need to deliver such genetic material in a safe and efficient way to target cells. In this sense, at a preclinical level, non-viral vectors for CRISPR/Cas delivery predominate over the use of viral vectors (70% versus 30%, respectively) [44]. Among non-viral gene delivery systems, we can differentiate the development of physical and chemical methods.

### 3.1. Physical Methods

Basically, physical methods are vector-free systems based on a controlled and reversible deformation of cytoplasmic membrane during short periods of time that allow the entry of genetic material on target cells [22]. Although highly effective, these methods are normally restricted to ex vivo gene therapy, which is mainly due to the challenge that represents the fine control of physical parameters that produce the formation of transient pores into the cellular membrane in vivo conditions. Such technological limitations can result not only in a loss of action but also in an increase of the cellular toxicity [45]. Transient pores on cellular and even nuclear membranes can be induced by the application of external electrical pulses, whose amplitude and duration are controlled, and depend on the particular characteristics of the target cell [22]. Pores can also be created if a cell´s membrane is mechanically deformed when cells are forced to pass through microfluidic-based channels which the diameter is smaller than that of the cell [46]. Other physical methods that also can be used to deliver genetic material into target cells efficiently include the direct microinjection of genetic material into cells by a micropipette, the induction of cellular uptake by stimulation of the macropinocytosis pathway with a hyperosmolar buffer containing sodium chloride and propanbetaine, or the hydynamic injection. This last technique consists of the quick injection of genetic material into the tail of rodents in volumes close to 10% of the total body weight [47].

### 3.2. Chemical Methods

**Figure 2:**
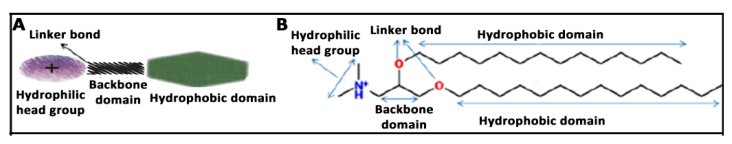
(**A**) General chemical structure of cationic lipids for gene therapy applications with four domains. (**B**) Chemical structure of cationic lipid 2,3-di(tetradecyloxy)propan-1-amine. The hydrophilic head domain consists on a protonated amine group, the backbone is a glycerol-based structure, the linker is an ether, and the hydrophobic domain consists of a double hydrocarbonated alkyl chain of 14 carbon atoms.

Although inorganic compounds such as magnetite, silica, or calcium phosphate, to name just a few ones, have shown great potential to shuttle genetic material, most chemical vectors are based on organic compounds such as cationic lipids or cationic polymers [48]. Amphiphilic cationic lipids for gene delivery applications normally share four domains in their chemical structure [49]: a hydrophilic polar head group, a hydrophobic apolar group, a linker, and a backbone. The positively charged hydrophilic polar head group interacts electrostatically with the negatively charged genetic material, obtaining the corresponding lipoplexes [50]. The composition of the hydrophobic apolar group can affect the relevant physicochemical and biological parameters that influence the transfection process such as the stability of the formulation, the DNA protection from nucleases, or the endosomal escape. The chemical composition of the linker domain influences both the flexibility and degradation of cationic lipids. Finally, the backbone group is the domain that separates the hydrophilic polar group from the hydrophobic apolar group, of which asymmetric glycerol-based backbone domains are the most commonly used for gene delivery purposes. A schematic representation of the general chemical structure of cationic lipids for gene therapy applications can be observed in Figure 2. Small changes in the chemical structure of any of the four domains can affect both the physicochemical and biological parameters that regulate the transfection process [51]. Normally, to enhance the transfection efficiency of cationic lipids, they are incorporated into vesicles made up of phospholipids resulting in corresponding liposomes [52], or solid lipid nanoparticles (SLNs) if the core of the nanoparticle is a solid lipid stabilized with surfactants [53]. Lipid nanoparticles have been used in the formulation of Patisiran^®^ to deliver siRNA genetic material in the liver after intravenous administration to suppress the production of transthyretin in hereditary transthyretin-mediated amyloidosis (hATTR) patients [54].

Apart from cationic lipids, cationic polymers with different physicochemical properties are also often used as gene delivery systems, obtaining the corresponding polyplexes after genetic material is adsorbed on their surface or entrapped into the polymeric matrix. Most of those cationic polymers include chitosans [55], polyethylenimine [56], or poly (*L*-lysine) [57]. Moreover, hybrid compounds, made by a combination of both polycationic and polyanionic polymers, and with different organic and inorganic materials such as polymers, magnetite, or lipids can be used also to deliver genetic material for different purposes [58,59,60,61,62].

In addition to classic chemical compounds, due to recent advances on nanotechnology and the interest in the development of non-viral vectors, other materials have recently emerged as promising gene delivery systems [48]. For instance, nanodiamonds (NDs) present fitting properties for gene delivery applications due to their high surface area-to-volume ratio, biocompatibility, scalability, and precise particle distribution [63]. Additionally, NDs can be easily functionalized to obtain hybrid compounds by electrostatic interactions with hydrophilic cationic polymers such as polyethylenimine [64,65], lysine [66], or polyallylamine hydrochloride [67]. Another strategy is to include cationic groups, i.e., silane-NH_2_ or polyamidoamine (PAMAM) [68], on the chemical structure of NDs by the formation of covalent bonds. Apart from NDs, graphene oxide (GO), a precursor of graphene, is another material that has been recently investigated for gene delivery applications. GO is a biocompatible material that is easy to synthetize, reproducible, and cheap. In addition, GO has high dispersibility in water, and it can be easily functionalized with different kinds of polymers such as polyethylene glycol (PEG) [69], polyethylenimine (PEI) [70], or chitosan [71].

## 4. Niosome Nanoparticles for Gene Delivery

Niosomes are non-ionic-based surfactant unilamellar or multilamellar vesicles with a bilayer structure that have been used for around 40 years as drug delivery systems for different applications with low toxicity and desired targeting properties [72]. As in the case of liposomes, hydrophilic heads are orientated toward an aqueous solution, whereas hydrophobic groups are orientate toward an organic solution, so both hydrophobic and hydrophilic drugs can be delivered by niosomes [73]. The main difference between both nanocarriers is that in the case of niosomes, the phospholipids of liposome vesicles have been substituted by non-ionic surfactants [74]. Compared to liposome counterparts, niosomes are recognized for their higher chemical and storage stability, due to the presence of non-ionic surfactants in their structure [75]. In addition, niosomes can be easily prepared at a low cost, and they are less toxic than liposomes due to the presence of non-ionic surfactants [76,77]. All these characteristics justify the research on niosomes as an interesting platform for gene delivery applications.

### 4.1. Components on Niosome Formulations

In addition to the non-ionic surfactant, which enhances the stability and is the main component of niosomes, other chemical compounds can be incorporated into the niosome vesicles such as cationic lipids that interact electrostatically with the negatively charged genetic material to obtain corresponding nioplexes at different cationic lipid/genetic material ratios and “helper” components that improve their biological performance [78]. Any slight modification of both the relationship and the chemical structure of these components can affect, in a significant way, the relevant physicochemical parameters of the formulation that regulate the transfection process such as the size, polydispersity index, and morphology [79].

Non-ionic surfactants can be classified into four different categories: alkyl ethers, alkyl esters, alkyl amides, and esters of fatty acids [73]. Some non-ionic surfactants that have been used in niosomes designed for gene delivery applications include polyoxyethylene alkyl ether (Brij^©^ [73]), polysorbates (Tween^©^ [80]), sorbitan fatty acid esters (Span^©^ [81]), or poloxamers [82]. The most relevant parameters of non-ionic surfactants to consider are the hydrophilic/lipophilic balance (HLB), which can be used as a “saving guide” parameter to select the appropriate surfactant [83], the critical packing parameter (CPP), which plays an important role in the vesicular-forming ability of niosomes [84], or the gel liquid transition temperature (T_C_), which has a relevant impact on the drug entrapped efficiency [85]. Among the cationic lipids, some of the most employed in the elaboration of niosomes for gene delivery purposes include 2,3-di(tetradecyloxy)propan-1-amine hydrochloride salt [86], 3β-[*N*-(dimethylaminoethane)-carbamoyl]-cholesterol hydrochloride salt (DC-Chol, [87]), *N*-[1-(2,3-Dioleoyloxy)propyl]-*N*,*N*,*N*-trimethylammonium methylsulfate salt (DOTAP, [88]), 1,2-di-*O*-octadecenyl-3-trimethylammonium propane chloride salt [83], or 1-(2-dimethylaminoethyl)-3-[2,3-di (tetradecoxy) propyl] urea [51], to name just a few. Any slight change of any of the four chemical domains of cationic lipids influences the transfection efficacy mediated by niosomes [49]. Regarding “helper” lipids, they are normally neutral components i.e., cholesterol, that when used in appropriate amounts enhance both the rigidity and the colloidal stability of formulations, promoting the gel liquid transition temperature of niosomes and the interactions with the apolar group of non-ionic surfactants [89]. In addition, they can affect biological parameters such as the cellular uptake and posterior intracellular trafficking of niosomes [90]. Squalene and squalane, which are natural lipids belonging to the terpenoid family, as well as biochemical precursors of the synthesis of cholesterol and other steroids, have also been incorporated in niosome formulations [90,91]. Another commonly used “additional” component on niosome vesicles is PEG. When niosomes “decorated” with hydrophilic PEG chains are administered into the bloodstream, the aqueous layer on the vesicular surface avoids endocytosis by the reticuloendothelial system (RES), and it therefore increases the half-live period of such niosomes in blood [92]. 

### 4.2. Niosome Preparation Methods

**Figure 3:**
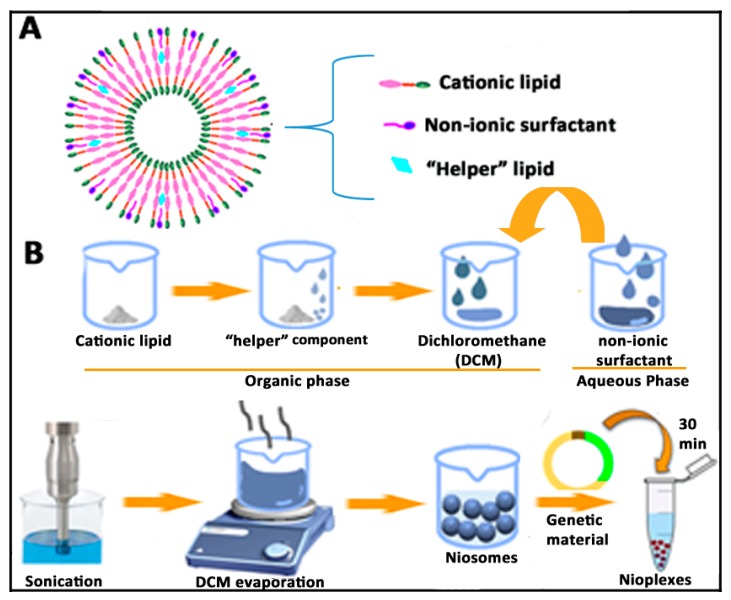
(**A**) Bilayer structure of niosomes and disposition of components. (**B**) Schematic representation of the solvent evaporation method for the elaboration of niosomes and corresponding nioplexes.

Niosomes can be easily elaborated by the solvent-evaporation method. Basically, both the cationic and “helper” lipids are dissolved in a small volume of organic phase, where the aqueous phase containing the non-ionic surfactant is added. After a brief sonication period, an emulsion is obtained. Such emulsion can be left under magnetic agitation to evaporate the organic solvent, which will produce the resuspension of the niosome vesicles into the aqueous phase [93]. A brief schematic representation of the niosome components and their elaboration by the solvent evaporation process can be observed in Figure 3.

The film-hydration method is basically a modification of the previously described solvent evaporation method, in which the organic solvent is evaporated in a round-bottomed flask using a rotatory vacuum evaporator. As a result, a dry film of lipids will be formed, which is thereafter hydrated with the aqueous phase above the transition temperature of the surfactant [94]. Another interesting and single-step alternative to elaborate niosomes without the use of organic solvents is the bubble method. In this case, large unilamellar niosomes vesicles can be obtained when both the surfactant and lipids are heated over 70 °C in a buffer solution. Then, the dispersion is mixed with a high shear homogenizer followed by the bubbling of nitrogen gas at 70 °C [73]. In addition to the bubble method, other alternative to obtain niosomes without the use of organic solvent is the lipid injection method. In this case, lipids and surfactants are melted and thereafter injected into an aqueous phase under heat and continuous agitation to get a final suspension of niosomes [95]. In order to obtain small unilamellar niosome vesicles, the microfluidization method can be used. In this case, niosomes are obtained when two fluidized streams pumped at specific speed interact with each other in small and specifically designed microchannels for fast mixing [95]. Interestingly, this technique allows the possibility of working in parallel with large volumes, which enhances the scalability of the production process [96]. The precise and detailed elaboration of niosomes by other different techniques such as trans-membrane pH gradient uptake, supercritical reverse phase evaporation, or the freeze and thaw process can be looked up in two excellent articles that have been recently published [73,85].

Once niosomes have been prepared using any of the previously above-mentioned techniques, the corresponding nioplexes can be obtained after the addition of a solution of the pertinent genetic material to the colloidal suspension of niosomes (Figure 3). Due to the electrostatic interactions between the positively charged amine groups of the cationic lipids incorporated into the niosome vesicles and the negatively charged phosphate groups of the genetic material, nioplexes can be easily obtained at different cationic lipid/genetic material ratios [76]. In the case the obtained niosomes are not going to be used soon, they can be stored at 4 °C during several weeks, without affecting the main physicochemical parameters that influence the gene delivery process [9].

### 4.3. Physicochemical Characterization of Niosome Nanoparticles

Normally, after niosome elaboration and before performing any biological experiments, some of the most relevant physicochemical properties involved in the nucleic acid delivery of niosomes are evaluated as a *screening* methodology to select the most suitable composition and concentration of the chemical components [78]. Dynamic light scattering (DLS) can evaluate the hydrodynamic diameter and the polydispersity index (PDI) of both niosomes and corresponding nioplexes in a Zetasizer instrument, while the morphology and distribution of niosome colloidal dispersions can be examined by different microscopic techniques [76]. The DLS technique is based on the random Brownian movement of small particles and the light scattered when a laser irradiates the colloidal suspension, which is highly dependent on the ion concentration [78]. The hydrodynamic diameter is usually obtained by cumulative analysis, which requires a narrow and monodisperse sample distribution, typically with PDI values below 0.5 for comparative purposes [97]. When genetic material binds to the surface of cationic niosomes at different cationic lipid/genetic material ratios, normally, the nioplexes size fluctuates slightly due to the condensation effect produced by the electrostatic interaction, which would decrease the size and the space demanded by the genetic material, which would increase in vesicular size [91]. Such slight alteration of the final size can affect the endocytosis pathway and consequently the posterior intracellular trafficking of niosomes [34,90,98]. In addition to size, the PDI value also changes upon the incorporation of genetic material on the surface of cationic niosomes. In this case, polydispersion typically increases due to the heterogeneous distribution of such genetic material [49]. Normally, niosomes exhibit spherical morphology that can be evaluated under transmission electron microscopy (TEM) and cryo-TEM [76]. These techniques can require the addition of staining agents, and they can also be used to evaluate the size and distribution of niosomes, although they may not correlate with DLS due the difference regarding the manipulation of the samples [91]. If the samples to analyze are in solid form, scanning electron microscopy (SEM) can also provide information about the morphology of niosomes. For a more precise analysis, for instance, to determine the characteristics of bilayers, scanning tunneling microscopy (STM) or small angle X-ray scattering (SAXS) techniques can be used [99,100].

The degree of the buffering capacity of cationic niosomes represents the potential of such non-viral vectors to escape from the degradation in the acidic compartment of lysosomes due to the incorporation of H^+^ into the lipid structure. Such buffering capacity can be measured by an acid–base titration assay [101]. Briefly, colloidal suspensions of niosome formulations are titrated with a solution of NaOH to reach a basic pH value. Next, niosomes are titrated again, but in this case with an HCl acid solution, to evaluate the capacity to absorb H^+^ when different volumes of the acid solution are added.

Another relevant parameter that can be determined to predict the stability of niosomes is the zeta potential (ζ). This value is related to the superficial charge of the formulation and can be obtained from a Zetasizer instrument by a laser Doppler velocimetry (LDV) technique [86]. Typically, it is accepted that ζ values of nanoparticles over 20 mV (either negative or positive) prevent aggregation by electrostatic repulsion [102]. When nioplexes are elaborated, positively charged cationic groups of niosomes are partially neutralized by the negatively charged phosphate groups of genetic material, resulting in a decrease of superficial charge which will depend on the cationic lipid/genetic material ratio [83]. Normally, nioplexes are elaborated at positive cationic lipid/genetic material ratios to enhance the cellular uptake of such nioplexes by interaction with the negatively charged cell membranes. Additionally, the interaction between the cationic lipids of niosomes and the genetic material can also be evaluated at a molecular level by isothermal titration calorimetry through the measurement of the heat released when such binding occurs [103]. Agarose gel electrophoresis assays can be used to evaluate the capacity of niosomes to condense, release, and protect the genetic material from enzymatic digestion [86]. In this sense, it is well established that a delicate balance between condensation and release capacity needs to be obtained at an appropriate cationic lipid/genetic material ratio to guarantee the condensation and protection efficiency, as well as the release of such genetic material to reach the nucleus of the target cell [91].

**Figure 4:**
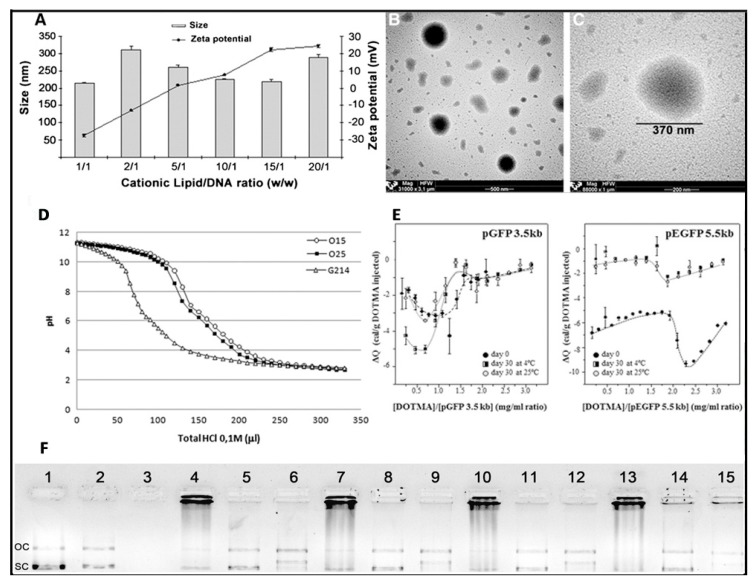
Physicochemical characterization of niosome formulations in terms of size, superficial charge (**A**), and morphology (**B**,**C**), adapted with permission from Puras et al. [91]. Buffering capacity assay (**D**) and isothermal titration calorimetry (**E**), adapted with permission from Agirre et al. [101]. Copyright 2019, Elsevier B.V and Gallego et al. [9]. Copyright 2014, Elsevier B.V. DNA binding capacity, release and protection from enzymatic digestion (**F**), adapted with permission from Puras et al. [91]. Copyright 2014, Elsevier B.V. OC, Open circular, SC supercoiled.

The stability of non-viral vectors is another issue that needs to be considered, due to its relevant effect not only on physicochemical parameters, but also on biological properties [85]. To evaluate the stability, some of the previously commented parameters such as particle size, PDI, or ζ value are monitored in different temperature and humidity conditions over time [104,105,106]. An interesting approach to enhance the physical stability of niosomes is to obtain a stable dry powder formulation by lyophilization that can later be resuspended in the appropriate solvent before use. In this case, the selection and the concentration of the cryoprotector, along with the parameters of the lyophilization process need to be evaluated to guarantee the stability of the dry powder [107,108]. The analysis of some of the previously described physicochemical parameters on a niosome formulation can be observed in Figure 4.

### 4.4. In Vitro Biological Evaluation of Niosomes for Gene Delivery

**Figure 5:**
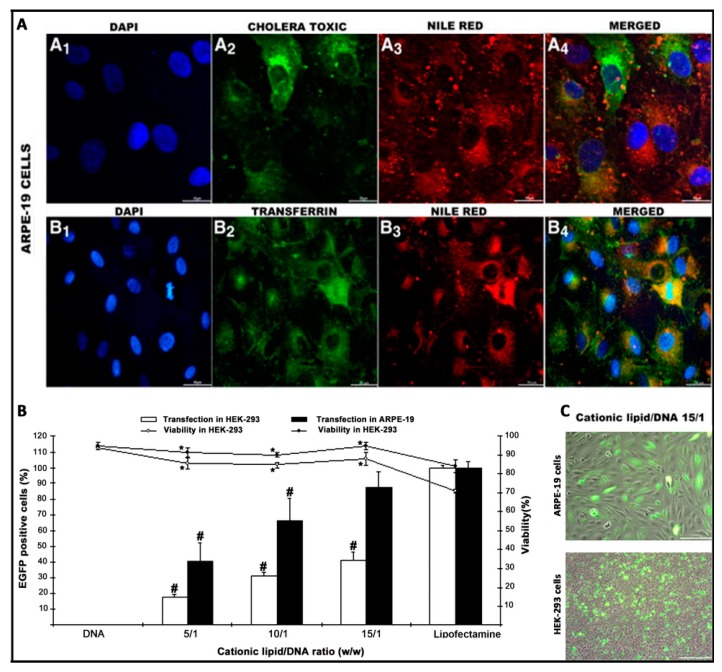
Biological evaluation of niosomes in terms of intracellular trafficking (**A**), transfection efficiency (**B**), and morphology (**C**), adapted with permission from Puras et al. [91]. Copyright 2014, Elsevier B.V.

Once the most relevant physicochemical parameters that affect the transfection process have been analyzed, and before performing any in vivo assay, several in vitro studies are normally performed to evaluate the toxicity and the efficiency of different formulations as a screening methodology of different candidate formulations. The cationic lipids in the structure of the niosome vesicles can destabilize the cell membrane, induce apoptosis, and therefore be toxic at high doses [109]. To minimize these effects, different strategies can be followed such as to increase the incorporation of the non-ionic surfactant, reduce the cationic lipid/genetic material ratio, or reduce the exposition time of nioplexes [49]. Since cell toxicity is mainly caused by the chemical structure of the cationic lipid and also has a clear cell-dependent effect, each application needs to be individually addressed [110]. Cell viability can be qualitatively evaluated by microscopy or can be quantified by flow cytometry with the use of appropriate fluorescent dyes such as propidium iodide or 7-Amino-actinomycin D (7-AAD), which penetrate into damaged cells [97]. Alternatively, colorimetric assays, such as CCK-8 (Cell Counting Kit-8) or MTT (3-[4,5-dimethylthiazole-2-yl]-2,5-diphenyltetrazolium bromide; succinate dehydrogenase activity), can be used to evaluate cell viability by absorbance [49]. Normally, dead cells are excluded from the transfection efficiency results. A common strategy to evaluate the initial transfection efficiency of niosomes is the use of reporter plasmids based on the luciferase of fluorescence [111,112] (Figure 5). It is worth mentioning that to get an optimal therapeutic effect, both the percentage of cells that incorporate the transgene, along with the amount of protein expression by transfected cells need to be considered. Although this approach can provide an overview of the gene delivery efficiency of niosomes, further readjustments on cationic lipid/genetic material ratio need to be performed when moving to therapeutic plasmids, since both the composition and size of the plasmid directly influence the transfection efficiency process [113].

The knowledge of the endocytosis pathway and the posterior intracellular trafficking to reach the nucleus of target cells can be useful to design more efficient and safer niosome vesicles for gene delivery applications [51]. For that purpose, specific fluorescent endocytic markers such as dextrans, cholera toxin B, or transferrin can be used to stain the most representative endocytosis pathways (macropinocitosis, caveolae, and clathrin-mediated endocytosis, respectively). The colocalization of such dyes with fluorescent labelled niosomes, or preferably, fluorescent plasmids attached on the surface of niosomes can be qualitatively evaluated by confocal microscopy [91], or quantified by different overlay coefficients, such as Mander´s or Pearson´s colocalization coefficients [9,114] (Figure 5). Additionally, intracellular trafficking studies can be completed with lysosome markers such as lysotrackers [90] or with different uptake inhibitors such as genistein, wortmannin, or chlorpromazine, to inhibit selectively caveolae, macropinocytosis, or clathrin-mediated endocytosis, respectively [115].

All of the previously mentioned in vitro studies can be performed as a proof of concept in Human Embryonic Kidney (HEK-293) culture cells, which is a one of the most employed models for transfection studies [116]. However, because transfection efficiency is a highly cell-dependent process, other cell lines that are more representative such as ARPE-19 or NT2 cells can be used for retinal and brain gene delivery purposes [91,93]. Additionally, and as a more realistic scenario that resembles in vivo conditions, primarily the culture cells of both retina and brain can be used [51]. When primarily culture cells are used, the transfection efficiency decreases considerably when compared to values obtained in immortalized cells lines. Therefore, primarily cultures are normally used to evaluate the kind of cells that have been transfected by immunohistochemistry rather that the transfection efficiency in quantitative terms [9,51]. In the case of immune-privileged organs such as the brain and eye, in addition to culture cells, different and sophisticated in vitro models based on microfluidic chips of both the BBB and BRB can be used to better mimic the in vivo conditions and predict their behavior performance [117,118].

## 5. Eye as Main Goal

The old concept of immune privilege appeared in 1948, when this term was applied to sites in the body where foreign tissue grafts can survive for extended periods of time, whereas similar grafts placed at a regular site in the body are acutely rejected [119]. This concept needs to be differentiated from the privileged immunity concept, which refers to the capacity of specific organs to select the most suitable and effective immune response to guarantee their proper functions in health and pathology [120]. In this review, we will refer to eye as an immune-privileged central nervous system (CNS) organ, in the sense that such organs are less likely to react against the inflammatory processes caused by foreign agents, because they are protected from the rest of the organism by the BBB [121].

### 5.1. General Concepts

The eye has been classically considered as an amenable organ to be targeted by in situ gene therapy [122]. Due to its reduced size and compartmentalized anatomy, small amounts of vector are required to get a satisfactory effect, and such vectors can be placed in close proximity to the target cells, rather than being systemically administered, which minimizes the potential adverse reactions [123]. Furthermore, due to the isolation from the rest of the organism, the risk of adverse effects is considerably reduced [124]. In addition, the visual function and retinal structure can be evaluated through non-invasive methods [125], and because most of the inherited retinal diseases are symmetric, the untreated eye can be used as control, reducing the number of experimental animals in the laboratory at a preclinical level [126].

The recently approved by FDA (Food and Drug Administration) and EMA (European Medicines Agency) Luxturna^®^ drug represents the most successful example of retinal gene therapy. Luxturna^®^ is indicated for the treatment of Leber congenital amaurosis type 2 (LCA2) disease due to bi-allelic mutations in the RPE65 gene expressed in RPE cells. With this gene therapy strategy, a functional copy of the required gene is delivered into the subretinal space by adenoasociated virus (AAV) vectors [127]. Although the results obtained by Luxturna^®^ offer an encouraging future for gene therapy applied in ophthalmology, the translation of such success to other retinal conditions will not be an easy task. AAV vectors target mainly the RPE layer, where the RPE65 gene codifies the required enzyme of the visual cycle. However, most of the genetic mutations of the retina affect the cells of the neuroretina, especially photoreceptors (PR), where AVV are not so efficient [128]. In addition, it has been reported that AAV virus can enter into the visual pathways of brain after subretinal injection, which rises major concerns due to the potential to trigger unexpected outcome effects [129]. In addition, AVV packing capacity is limited to approximately 4.7 kb, which jeopardizes its use to deliver genes with larger coding sequences i.e., ABCA4, MYO7A, or CEP290 for the treatment of relevant pathologies of the retina such as Stargardt disease, Usher syndrome type 1B, or LCA type 10, respectively [122]. Another relevant concern is the high cost of AVV–based Luxturna^®^ treatment, which is around $850.000 per-patient in the U.S., which makes it difficult for affected patients to access to such innovate treatments. Therefore, it looks logical to explore other safer and cheaper alternatives to deliver genetic material into the retina. In this sense, non-viral vectors based on cationic niosomes have recently shown promising results at a preclinical level.

### 5.2. Niosomes for Gene Delivery to the Retina

The first evidence of niosome vesicles as efficient gene delivery systems into the retina was reported in 2014, when niosomes based on the 2,3-di(tetradecyloxy)propan-1-amine cationic lipid, combined with the squalene “helper” lipid and polysorbate 80 non-ionic surfactant were able to deliver in a safe and efficient way the reported EGFP (Enhanced Green Fluorescent Protein) plasmid to the rat retina after both intravitreal and subretinal administrations [91]. Previously, it was reported that the aforementioned cationic lipid was able to silence gene expression upon covalent conjugation with RNA molecules [130], and that corresponding lipoplexes transfected efficiently RPE and some PR cells after subretinal injection [50]. These data reflect the suitability of such cationic lipids to be used for gene delivery purposes and its inclusion in a novel niosome formulation where non-ionic surfactant polysorbate 80 was incorporated to enhance the stability of vesicles. In addition, squalene, a natural lipid belonging to the terpenoid family, was also incorporated as a “helper” lipid due to the promising transfection results obtained previously with this compound in other cationic lipid emulsions [131,132]. Nioplexes around 200 nm and +25 mV were obtained upon addition of the reported pCMS-EGFP plasmid at a 15/1 cationic lipid/DNA ratio. Such nioplexes entered mainly by the clathrin-mediated endocytosis pathway in cultured RPE cells, and immunohistochemistry studies reflected that nioplexes were able to deliver the reported plasmid into different layers of the retina, depending on the administration route. Interestingly, protein expression was still observed 28 days after both subretinal and intravitreal injections.

The following year, in 2015, a ternary non-viral vector based on protamine/DNA/niosome expressed locally the EGFP protein in PR close to the in situ subretinal administration, and a more uniform distribution of the protein expression was observed in the inner layers of the retina, especially in ganglion cells, after intravitreal injection. As in the previous study, protein expression persisted for at least one month after both administrations [86]. Protamine is an FDA-approved small peptide obtained from the sperm of herring and salmon that efficiently condenses DNA due to its positive charge. Arginine sequences on protamine promote the nuclear import of genetic material, which is especially relevant in slow-dividing retinal cells [133]. However, the high hydrosolubility of protamine hinders the interaction with lipophilic membrane cells [134]. Consequently, to enhance such interaction, protamine was incorporated in lipid formulations such as SLNs [135] or liposomes [136] but not in niosomes for retinal gene delivery. The incorporation of protamine in ternary vectors (protamine/DNA/niosomes, at 1:1:5 ratios, respectively) reduced the size to 150 nm and enhanced DNA condensation capacity. Interestingly, it also reduced the amount of cationic lipid required to transfect the rat retina, and therefore, increased cell viability.

Since small modifications on the chemical structure of the components in the cationic niosomes can affect the gene delivery capacity, in 2016, the transfection efficiency of three different cationic lipids was evaluated in rat retina [51]. Such cationic lipids shared the same hydrophobic tail and the same glycerol-based building block, differing only among them on the polar head formed by an amino group, a glycine triglycine, and a dimethylaminoethyl group. Both squalene and polysorbate 80 were used as “helper” lipids and non-ionic surfactants, respectively. After an extensive physicochemical characterization and in vitro evaluation in different cell lines, the results showed that nioplexes based on the cationic lipid that had the dimethylaminoethyl structure on the polar head group were the most efficient for retinal gene delivery. After the intravitreal injection of nioplexes at a 30/1 cationic lipid/DNA ratio, EGFP expression was uniformly distributed, overall, in the ganglion cell layer. The PEG chains on the polysorbate 80 non-ionic surfactant could prevent the aggregations of cationic niosomes with negatively charged components, glycosamineglycanes, and fibrilar structures present in the vitreous, enhancing therefore the diffusion through the vitreous humor and the transfection efficiency [137]. Interestingly, after intravitreal injection, protein expression was also detected in some outer cells of the retina. The transfection of PR and RPE cells by intravitreal injection instead of subretinal injection represent a great challenge to face genetic pathologies that affect the outer segments of the retina, avoiding the harm of sensitive neuronal tissue that is classically associated to subretinal injection [138].

As in the case of squalene, lycopene is another natural terpenoid compound found at high concentration levels in the eye, which is classically known by its biological properties as an antioxidant agent, cytoprotector, and immunomodulator, among many others [139]. Therefore, in 2017, lycopene-based niosome formulations were elaborated by the solvent evaporation technique and evaluated for retinal gene delivery capacity prior to physicochemical and biological characterization [140]. The resulting nioplexes at an 18/1 mass ratio showed nanometric size with low polydispersion, spherical shape, positive superficial charge, and the capacity to stabilize DNA against enzymatic degradation. The cellular uptake of such nioplexes was mediated mainly by the macropynocitosis and caveolae pathways. After intravitreal injection, the outer segments of the retina were also efficiently transfected in a safe way.

As the main component of a niosome vesicle is the non-ionic surfactant, it looks logical to study the influence of different non-ionic surfactants in the design of niosomes for retinal gene delivery applications. In fact, in 2018, three niosome formulations that only differed in the polysorbate non-ionic tensioactive were elaborated by the solvent evaporation technique. The three niosome vesicles shared the same commercially available cationic lipid 1,2-di-O-octadecenyl-3-trimethylammonium propane (DOTMA), the same “helper” lipid squalene, and had polysorbate 20, polysorbate 80 or polysorbate 85 as non-ionic tensioactives [83]. Polysorbate 20 has the highest HLB value (16.7) and upon the incorporation of reported pCMSEGFP plasmid on the surface of corresponding niosomes to obtain nioplexes at a 2/1 ratio of cationic lipid/DNA, RPE cultured cells were successfully transfected without signs of toxicity. Intracellular trafficking studies showed that the hydrophilic nature of the polysorbate 20 non-ionic surfactant promoted caveolae-mediated endocytosis and evaded colocalization with the lysosome compartment, which to some extent could explain the difference observed in transfection efficiency among the three niosome formulations. In the primary culture cells of retina, such formulations were well tolerated, in contrast to the commercially available Lipofectamine 2000^TM^, and they expressed the fluorescent protein mainly in glial cells. After in situ subretinal injection, protein expression was observed mostly in RPE cells and also in the inner layers of the retina, whereas intravitreal injection transfected overall the ganglion cell layer.

However, the success of gene therapy does not only rely on the composition of the gene delivery system. In fact, vectors are only one part of the complex formulation. The other half is conditioned by the characteristics of the genetic material. In this sense, in 2019, the same cationic niosome formulation and three different GFP-encoding genetic materials consisting of minicircle (2.3 kb), its parental plasmid (3.5 kb), and a larger plasmid (5.5 kb) were combined to form nioplexes. Obtained results showed that the lack of unmethylated CpG regions in the mcDNA rendered nioplexes with better physicochemical properties, stability, cell tolerance, and transfection efficiency in different layers of the rat retina after both intravitreal and subretinal injections, which reinforce the importance of the genetic material size and composition in the design of gene therapy vectors.

**Figure 6:**
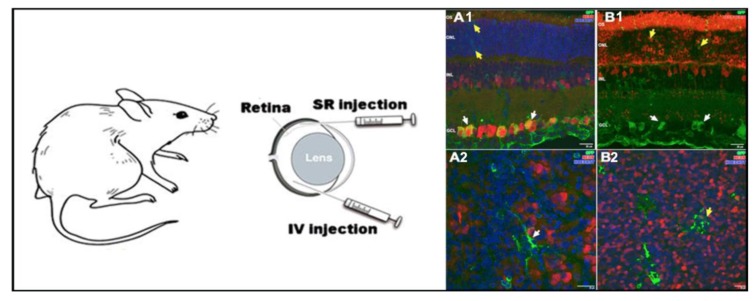
In vivo applications of niosomes in retina. Niosomes transfected different layers in the rat retina, depending on the administration route. Retinal cross sections micrographs obtained by confocal microscopy (**A1**,**B1**), confocal fluorescence micrographs of whole mount (**A2**,**B2**). Adapted with permission from Mashal et al. [114]. Copyright 2019, Elsevier B.V.

Taking into account that cationic niosomes represent a tunable platform for retinal gene delivery applications, in 2019, chloroquine was incorporated as a “helper” component into cationic niosomes based on the 2,3-di(tetradecyloxy)propan-1-amine (hydrochloride salt) cationic lipid, and a mixture of both poloxamer 188 and polysorbate 80 as non-ionic surfactants [115]. Chloroquine is a 4-aminoquinoline drug with promising properties for retinal gene therapy, since it can cross the BRB, interacts with negatively charged DNA molecules, and also promotes endosomal scape [141]. However, its clinical application is limited due to the high toxicity exhibited [142]. Therefore, chloroquine was incorporated within a niosome formulation to keep its gene delivery properties at low doses, reducing its toxic effect. At a 10/1 cationic lipid/DNA ratio, the resulting chloroquine concentration was only 25 μg, and it did not induce any significant cytotoxicity. In contrast, protein expression through different layers of the retina was increased as can be observed in Figure 6.

## 6. Brain as Main Goal

As in the case of vision, brain functions are essential for survival. Therefore, sophisticated mechanisms have been developed over many years of evolution to protect and isolate such sensitive organs with limited regeneration capacity from potentially damaging effects [31]. In the case of the brain, the tightly joined endothelial cells of BBB are impermeable for almost 100% of macromolecular and over 98% of small molecular drugs [143]. The transport of essential nutrients such as amino acids and glucose is mediated by specific receptors present in the BBB [31]. Although recent advances on gene therapy offer reasonable hope to face some devastating pathologies that affect relevant CNS organs such as the brain and eye, the isolation of those organs from the rest of the organism by the BBB and BEB prevents delivery systems from crossing such hurdles [43,144]. Consequently, effective gene therapy approaches to treat both inherited and acquired diseases of brain rely on the in situ administration of genetic material by invasive routes [43]. Such cumbersome gene delivery strategy can jeopardize the acceptance of treatments and increase aftercare cost due to the treatment of related side effects [145].

### 6.1. General Concepts

Gene therapy has shown great progress in clinical trials over the last decade to face both inherited and acquired devastating brain diseases that do not have a reasonably effective treatment with conventional drugs (https://alliancerm.org/publication/q2-2019-data-report). In the case of inborn metabolism mutations of one gene that affect the brain, such as mucopolysaccharidoses or Canavan disease, the approach normally consists of the delivery of a functional copy of the gene to restore the normal phenothype [146,147,148]. However, in the case of brain-acquired diseases, where more than one gene can be affected, such as brain cancer, Alzheimer’s, or Parkinson’s diseases, the genetic approach is more challenging, since the molecular basis of those disorders are still not understood [148,149,150]. BBB hampers the entry of gene expression vectors into the brain; consequently, gene treatments must be given after an invasive craniotomy, which in many cases jeopardizes the acceptance of patients enrolled in clinical trials due to the cumbersome approach and related side effects that increase the after-care cost as a consequence of additional hospital visits [151]. Moreover, because of the low diffusion of genetic material after in situ brain administration by craniotomy, few cells can be targeted by vectors, which prevent the access of the genetic material to the rest of the brain cells [152].

Apart from the route of administration, another confounding factor in brain gene therapy is the challenge that represents the delivery of genetic material into neurons. Brain neurons show strict organization to form complex neuronal networks, limited regenerative capacity, and low division rate, which hampers the entry of exogenous DNA into the nucleus [153]. Additionally, the molecular bases of many neurological disorders that affect the brain are still not understood.

Previously commented limiting factors, along with the need to use appropriate vehicles that are able to deliver efficiently the genetic material, justify the hard path of brain gene therapy to reach clinical practice. In fact, although many phase I clinical trials have been reported; only a few have reached phase II [154]. However, with the new emerging technologies applied to gene therapy, the cure of brain diseases might look like a reasonable option in the near future [155]. One of the most promising approaches is to explore the use of non-viral vectors as gene delivery systems due to their safer profile, easy production capacity, and lower cost when compared to their viral vector counterparts [154].

### 6.2. Niosomes for Gene Delivery to the Brain

**Figure 7:**
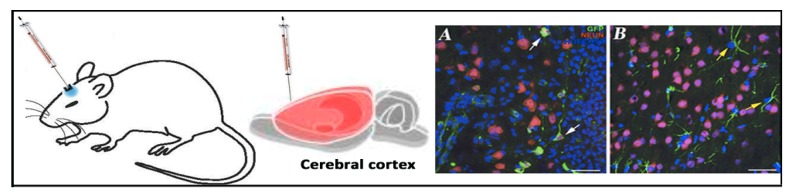
In vivo applications of niosomes in brain. (**A**) White arrows in the right side indicated identified neurons (red) that express EGFP (green). (**B**) Non-neuron cells (NeuN−) with glia morphology that express EGFP (green) were indicated by yellow arrows. Adapted with permission from Ojeda et al. [51]. Copyright 2017, Elsevier B.V.

Among non-viral vectors, niosomes based on cationic lipid 1-(2-dimethylaminoethyl)-3-[2,3-di (tetradecoxy) propyl] urea, combined with squalene as a “helper” lipid and polysorbate 80 non-ionic surfactant, were recently able to transfect the rat cerebral cortex after in situ administration [51]. Such cationic niosomes were elaborated by the solvent evaporation technique and exhibited a diameter of 200 nm with a low PDI value (0.21) and positive superficial charge over 30 mV. Physicochemical parameters were maintained after 100 days when formulations were stored at 4 °C. Upon the addition of reporter pCMSEGFP plasmid in their surface by electrostatic interactions at a 30/1 cationic lipid/DNA ratio, the resulting nioplexes were able to transfect efficiently both neurons and non-neuron cells in primary cultures obtained from the cortex of rat embryos, as revealed by immunohistochemistry studies as can be observed in Figure 7.

In 2018, another niosome formulation elaborated with a commercially available DOTMA cationic lipid, lycopene as a “helper” lipid and polysorbate 60 as a non-ionic surfactant, exhibited high levels of protein expression in both primary cortical cultures of rat embryos and in in vivo conditions after intracranial injection in the cortex [114]. Such niosomes were characterized in terms of size, superficial charge, polydispersity, or capacity to protect genetic material against enzymatic digestion in an agarose gel electrophoresis assay. After physicochemical characterization, in vitro studies were performed in human neuronal precursors NT2 cells. NT2 cells are considered as an attractive model to evaluate CNS gene delivery efficiency, which is mainly due to their potential capacity to be easily differentiated into both glial and neuronal cells, upon exposition to retinoic acid [93,156]. Additionally, genetically modified NT2 cells can be transplanted into CNS and migrate to specific regions of brain, acting as an interesting cell-based gene delivery platform to repair brain damages [157]. After 24 h post-transfection, nioplexes formulated at a 14/1 cationic lipid/DNA ratio exhibited approximately half the transfection capacity of the commercially available Lipofectamine^®^ 2000 but higher cell viability. Intracellular trafficking studies performed with both specific dye markers and blockers of most representative endocytosis pathways revealed higher cellular internalization by both caveolae and clathrin-mediated endocytosis than by macropinocytosis. In addition, buffering capacity studies revealed endosomal properties that could explain, at least in part, the high transfection efficiency observed in NT2 cells. Next, and before brain administration into the rat cerebral cortex, the primary cortical cultures of rat embryos were exposed to nioplexes in order to better mimic the in vivo conditions. In this scenario, NeuN^-^ cells with gial morphology expressed the protein, which was probably due to their phagocytic and mitotic activity [158]. The lack of protein expression into neurons was confirmed after the in situ intracranial injection of nioplexes. Again, only NeuN^-^ cells (neuroglia and cells in blood vessel wall) expressed the protein. In any case, although this niosome formulation failed to transfect brain neurons after intracranial injection, high levels of protein expression in glial cells suggest its possible application into CNS in glia-related neurological disorders such as epilepsy, Alzheimer’s, or Parkinson’s diseases, to name some of the most representatives ones [159].

Considering the previously reported properties of poloxamer 188 regarding biocompatibility and capacity to protect neurons against brain injury [160], its incorporation into niosomes based on the hydrochloride salt of 2,3-di(tetradecyloxy)propan-1-amine cationic lipid and polysorbate 80 surfactant was evaluated [82]. Therefore, two niosome formulations that differed only regarding the presence or absence of the non-ionic surfactant poloxamer 188 were elaborated by the reverse-phase evaporation technique and characterized to deliver the genetic material into the rat brain cortex. When poloxamer 188 was incorporated into the niosomes, the sizes of niosomes increased, which was probably due to the high HLB value of poloxamer 188 and to the interaction with the cationic lipid [161]. However, no significant change in zeta potential was reported, being over +40 mV. Agarose gel electrophoresis assays revealed that out of all the cationic lipid/DNA ratios studied, nioplexes based on niosome formulated with both non-ionic surfactants polysorbate 80 and poloxamer 188 at equal mass ratios protected the genetic material from enzymatic degradation. However, in the case of niosomes formulated only with polysorbate 80 as a non-ionic surfactant, at low cationic lipid/DNA ratios, the genetic material was degraded, which was probably due to the negative zeta potential value of those nioplexes that were able to condense but not protect the DNA. In vitro experiments on NT2 cells revealed that the addition of poloxamer 188 to niosomes enhanced the cell viability and the cellular uptake mediated by caveolae and macropinocytosis, which could explain the higher transfection values observed. In primary cortical cultures of rat embryos, gene expression was mainly observed in neurons with no signs of toxicity. However, when moving to in situ intracranial administration, nioplexes transfected glial cells but not the neurons of the cortex. Such in vitro and in vivo discrepancy could be explained by the different gene delivery mechanism in both biological scenarios.

Interestingly, the following year, in 2019, the same niosome formulation based on the 2,3-di(tetradecyloxy)propan-1-amine cationic lipid and a mixture at equal weight ratios of both polysorbate 80 and poloxamer 188 non-ionic surfactant was able to deliver the pUNO1-hBMP7 plasmid into NT2 (NTera2/D1 teratocarcinoma-derived) cells [162]. The human bone morphogenetic protein 7 (hBMP7) belongs to the transforming growth factor β superfamily, and its role is relevant for the development of bone, kidney, and nervous tissues [163]. At a 6/1 cationic lipid/DNA ratio, a significant release of hBMP7 (5.7 ng/mL) was detected in the supernatants of transfected cells, with no signs of toxicity. Next, the tumor-suppressive effect of hBMP7-expressing NT2 cells was investigated on the glioma cell line C6 in a transwell indirect co-culture system to avoid the drawbacks of direct co-culture system. In vitro co-culture results showed that the BMP7-overexpressing NT2 cells hampered the migration of C6 glioma cells, which highlights the potential of NT2 cell-based delivery of hBMP7 for impeding the metastasis of glioma cells [162].

## 7. Future Perspectives

After more than three decades of hard work, with normal ups and downs, nowadays, gene therapy represents a real and revolutionary clinical option, not only to treat but also to cure the molecular basis of some serious disease. In any case, and despite the promising future that awaits gene therapy, some controversial issues still need to be improved. At the moment, all of the gene therapy treatments approved for human use by different regulatory authorities are based on viral vectors, which rises controversy regarding their safety profile, limited gene-packing capacity, large-scale production, and high costs [164]. Consequently, during the last years, research on non-viral vectors has gained “momentum” as a safer alternative to their viral vectors counterparts, and the number of clinical trials has considerably increased since 2010 (www.clinicaltrials.gov).

**Figure 8:**
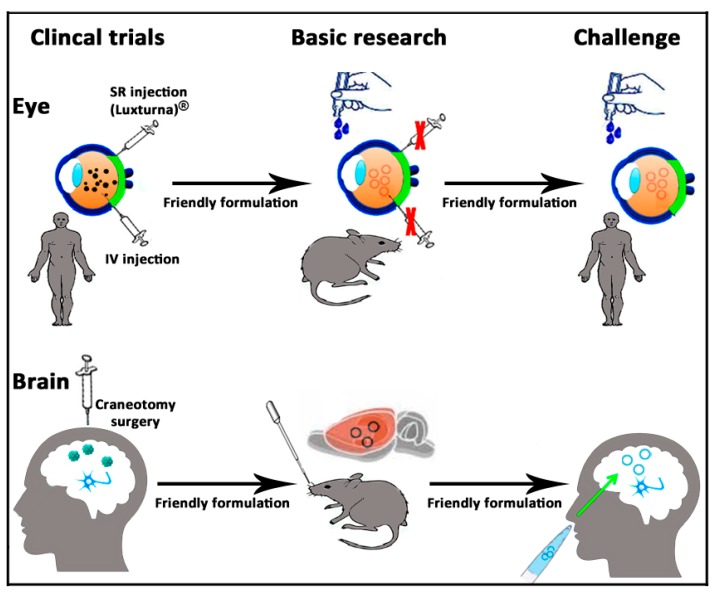
Schematic representation of a gene therapy approach based on non-viral vectors and non-invasive administration route to face brain and eye diseases.

In the case of eye and brain, which according to the old immune-privileged concept are organs isolated and protected from the systemic immune response of the organism [119], the use of less immunogenic non-viral vectors [165] is even more relevant to avoid damage in such sensitive organs [43]. In addition, in order to develop a more friendly approach, at a preclinical level, many nanotechnology-based formulations of different materials, shapes, and compositions can be tailored with specific ligands to overcome both BRB and BBB and deliver their cargo by non-invasive routes of administration such as topical instillation on ocular [166,167,168,169] and nose surfaces [170,171] (Figure 8). Considering the versatility of the cationic niosome platform for gene delivery applications, some of the biomaterials commonly used to overcome the BRB and BBB, such as transferrin, Annexin V, insulin, or gemini surfactants, could be incorporated in novel niosome vesicles, bearing in mind the recent results reported after the in situ administration of such non-viral vectors in both retina and brain cortex tissues.

Although it is possible to reach the brain cortex and retina in small animals at a preclinical level by non-invasive routes of administration, in order to reach the regular clinical practice, enough gene expression should be reached selectively in the specific target cells of those tissues in larger species, avoiding the distribution of the delivered gene in other tissues. At present, non-invasive approaches to reach both the brain cortex and retina require multiple administration doses at high concentrations, which enhance systemic absorption, and therefore, the appearance of unwanted effects in other tissues. In this sense, the use of cell-type specific promoters, inducible promoters, or the rapamycin regulation system offer a reasonable option to confine gene expression only in specific cell types, which avoids off-target effects in other cells [153]. Hence, the future direction to design non-viral vectors based on novel cationic niosomes to face inherited and acquired diseases of eye and brain by non-invasive routes of administration requires not only the use of appropriate biomaterials and targeting ligands coupled to the niosome platform but also the selection of the appropriate genetic material with its intrinsic characteristics. In any case, it is clear that such ambitious goals need to be addressed by a multidisciplinary approach. In this sense, the design of novel smart imaging and sensing catheter devices for surgical interventions based on next-generation technologies at the point of intervention would also minimize both the damage and cost occasioned by the in situ administration of gene therapy-based drugs in the brain and retina.

## Figures and Tables

**Table 1:** Gene therapy-based drugs on market for in vivo human use, including synthetic oligonucleotides.

Year (Agency)	Name	Indication	Genetic Material	Administration	Delivery System
2003 (FDA, China)	Gendicine	Head and neck squamous cell carcinoma	Bacterial plasmid	Intratumoral injection	Adenovirus
2004 (FDA, USA)	Macugen	Age-related macular degeneration	Synthetic aptamer	Intravitreal Injection	-
2005 (FDA, China)	Oncorine	Nasopharyngeal cancer	Viral DNA	Intratumoral injection	Adenovirus
2010 (FDA, USA)	Rexin-G	Meteastatic pancreatic cancer	Viral RNA	Intravenous infusion	Retrovirus
2012 (Russian ministry of Healthcare)	Neovasculgen	Atherosclerotic peripheral arterial disease	Bacterial plasmid	Intramuscular injection	-
2013 (FDA, USA)	Kynamro (Mipomersen)	Homozygous familial hypercholesterolemia	Synthetic ASO	Subcutaneous injection	-
2016 (FDA, EMA)	Imylgic	Multiple solid tumors	Viral DNA	Intratumoral injection	Oncolytic Herpex simple Virus
2016 (FDA, EMA)	Exondys 51 (Eteplirsen)	Duchene muscular dystrophy	Synthetic ASO	Intravenous infusion	-
2016 (FDA, EMA)	Spinraza (Nusinersen)	Spinal muscular atrophy	Synthetic ASO	Intrathecal administration	-
2016 (FDA, EMA)	Defibrotide	Veno-occlusive disease of liver	Single-stranded oligodeoxyribo nucleotides	Intravenous infusion	-
2018 (FDA, EMA)	Patisiran (Onpattro)	Familial amyloid polyneuropathy	RNA interference	Intravenous perfusion	Lipid nanoparticle
2018 (FDA, EMA)	Luxturna	Leber congenital amaurosis type 2	RPE 65 plasmid	Subretinal injection	Adeno-associated Virus
2018 (FDA)	Tegsedi (Inotersen)	Transthyretin-mediated amyloidosis	Synthetic ASO	Subcutaneous injection	-
2019 (FDA)	Givlaari (Givosiran)	Acute hepatic porphiria	RNA interference	Subcutaneous injection	-
2019 (EMA)	Waylivra (Volanesorsen)	Familial chylomicronemia syndrome (FCS)	Synthetic ASO	Subcutaneous injection	-
2019 (FDA)	Vyondys 53 (Golodirsen)	Duchene muscular dystrophy	Synthetic ASO	Intravenous injection	-
2019 (FDA)	Zolgensma	Spinal muscular atrophy	Bacterial plasmid	Intravenous infusion	Adeno-associated Virus
